# Cancer

**Published:** 1844-09-07

**Authors:** 


CANCER.

The treatment of this disease is null at the present period. Surgeons operate; and when patients refuse to submit to the operation, they tell them that there is nothing else for it. However, all agree that the operation does not cure; it does not even prolong life. Subjoined is the proof:—

From a return addressed to the Academy of Sciences by M. Leroy d'Etoilles, it appears that out of 1192 patients who had not been cut, 18 lived more than thirty years from the commencement of the disease; while of 801 who had been operated upon, 4 only were living after the same lapse of time.

There survived from twenty to thirty years after the development of the disease, 18 operated, 34 not operated; from six to twenty years, 88 operated, and 228 who had not undergone any operation.—*Tanchou in Comptes Rendus.*

